# The association of insertions/deletions (INDELs) and variable number tandem repeats (VNTRs) with obesity and its related traits and complications

**DOI:** 10.1186/s40101-017-0142-x

**Published:** 2017-06-14

**Authors:** Yee-How Say

**Affiliations:** Department of Biomedical Science, Faculty of Science, Universiti Tunku Abdul Rahman (UTAR) Kampar Campus, Jalan Universiti, Bandar Barat, 31900 Kampar, Perak Malaysia

**Keywords:** Indels, Variable number of tandem repeats, Genetic variation, Obesity, Anthropometric measurements

## Abstract

**Background:**

Despite the fact that insertions/deletions (INDELs) are the second most common type of genetic variations and variable number tandem repeats (VNTRs) represent a large portion of the human genome, they have received far less attention than single nucleotide polymorphisms (SNPs) and larger forms of structural variation like copy number variations (CNVs), especially in genome-wide association studies (GWAS) of complex diseases like polygenic obesity. This is exemplified by the vast amount of review papers on the role of SNPs and CNVs in obesity, its related traits (like anthropometric measurements, biochemical variables, and eating behavior), and its related complications (like hypertension, hypertriglyceridemia, hypercholesterolemia, and insulin resistance—collectively known as metabolic syndrome). Hence, this paper reviews the types of INDELs and VNTRs that have been studied for association with obesity and its related traits and complications.

**Main body of the abstract:**

These INDELs and VNTRs could be found in the obesity loci or genes from the earliest GWAS and candidate gene association studies, like *FTO*, genes in the leptin–proopiomelanocortin pathway, and *UCP2*/*3*. Given the important role of the brain serotonergic and dopaminergic reward system in obesity susceptibility, the association of INDELs and VNTRs in these neurotransmitters’ metabolism and transport genes with obesity is also reviewed. Next, the role of *INS* VNTR in obesity and its related traits is questionable, since recent large-scale studies failed to replicate the earlier positive associations. As obesity results in chronic low-grade inflammation of the adipose tissue, the proinflammatory cytokine gene *IL1RA* and anti-inflammatory cytokine gene *IL4* have VNTRs that are implicated in obesity. A systemic proinflammatory state in combination with activation of the renin–angiotensin system and decreased nitric oxide bioavailability as found in obesity leads to endothelial dysfunction. This explains why VNTR and INDEL in *eNOS* and *ACE*, respectively, could be predisposing factors of obesity. Finally, two novel genes, *DOCK5* and *PER3*, which are involved in the regulation of the Akt/MAPK pathway and circadian rhythm, respectively, have VNTRs and INDEL that might be associated with obesity.

**Short conclusion:**

In conclusion, INDELs and VNTRs could have important functional consequences in the pathophysiology of obesity, and research on them should be continued to facilitate obesity prediction, prevention, and treatment.

## Background

### Obesity and its genetic factors

The worldwide prevalence of overweight and obesity, which rose by 27.5% in adults and 47.1% in children between 1980 and 2013 [1], has been described as a global pandemic. Obesity, defined as abnormal or excessive fat accumulation that presents a risk to health, is associated with increased morbidity and mortality related to several conditions such as type 2 diabetes (T2D) and cardiovascular disease [2]. While obesity is mainly caused by environmental factors such as dietary habits and physical activity, it still has a strong genetic component. Family, twin, and adoption studies indicate that genetics account for 20–84% of population variation in body mass index (BMI) [3, 4], the most commonly used anthropometric measurement of adiposity apart from direct body fat content imaging techniques. Other anthropometric measurements such as waist circumference (WC) and waist–hip ratio (WHR) are more correlated with abdominal fat deposition (central adiposity) than BMI and are considered strong risk factors for T2D [5].

The study of genetic factors involved in body weight regulation in humans heavily extrapolated from studies on rodent monogenic obesity models in the 1990s. Monogenic obesity is a rare form of severe obesity that results from gene mutations that have large effect sizes. By screening children with severe, young-onset obesity for the genetic defects identified in mice, loss-of-function (LOF) mutations causing deficiencies are found in genes encoding for appetite-regulating hormones or their receptors such as leptin (*LEP*) [6], leptin receptor (*LEPR*) [7], proopiomelanocortin (*POMC*) [8], and melanocortin 4 receptor (*MC4R*) [9]. As these LOF variants are only found in monogenic obesity, they are uncommon in the general population. Therefore, various genetic approaches like candidate gene association studies and genome-wide linkage studies have been performed to identify susceptibility genetic loci for common polygenic obesity instead in the early 2000s. However, these approaches have been met with little success as the genes or loci found to be associated with obesity vary heavily across ethnic populations and are therefore difficult to replicate due to several reasons as reviewed in [10].

Fortunately, after the completion of the Human Genome Project in 2003 [11] and the International HapMap Project (Phase I) in 2005 [12] in conjunction with the development of high-throughput genotyping techniques as well as bioinformatics and statistical methods, various obesity genome-wide association studies (GWAS) were conducted from 2006 [13]. In GWAS, thousands of genetic variants are genotyped on a single microarray technology for association with obesity and its related traits and complications [14]. In the recent decade, numerous GWAS among Caucasians (like the Genetic Investigation of Anthropometric Traits (GIANT) consortium) [15], East Asians, and Africans, each analyzing >50,000 individual subjects, have identified >90 loci for obesity (reviewed in [16]).

### The potential of INDELs and VNTRs in obesity

Genetic variations in the human genome present as (i) single nucleotide polymorphisms (SNPs), (ii) insertions/deletions (INDELs) ranging from 1 to 10,000 bp in length [17], and (iii) structural variations that account for a greater number of base pairs. An example of structural variation is variable number tandem repeat (VNTR), which is a DNA sequence motif that is repeated several times in the genome continuously and constitutes Mendelian inheritance (reviewed in [18, 19]). VNTRs cover both microsatellites or short tandem repeat (STR) (1–6-bp-long motifs) [20] and minisatellites (hundreds of base pairs block motifs) [21, 22]. The number of times the sequence is repeated differs within and between individuals. The highly polymorphic nature of VNTRs makes them very informative as a class of markers to map for disease loci in family linkage studies [18].

SNPs are the most common type of genetic variations found in the human genome, and a substantial amount of research, such as that by the International HapMap Project, has been focused on accurately mapping and identifying them [23]. In spite of being the second most common type of genetic variations and constituting a large portion of the human genome [24], INDELs and VNTRs are more challenging to identify compared to SNPs, due to library preparation, sequencing biases, and algorithm artifact issues (reviewed in [25, 26]). Due to the nature of INDELs and VNTRs, genotyping them on the array platforms that SNP genotyping utilizes has led to technical problems like complications in scoring the number of repeats present. Unlike the vast amount of reviews available in the literature on the association of SNPs and larger forms of structural variation like copy number variations (CNVs) with obesity and its related traits (like anthropometric measurements, biochemical variables, and eating behavior) and its related complications (like hypertension, hypertriglyceridemia, hypercholesterolemia, and insulin resistance—collectively known as metabolic syndrome), INDELs and VNTRs have received far less coverage and attention. In fact, INDELs and VNTRs could have important functional consequences by regulating gene transcription and messenger RNA (mRNA) translation or by modifying the structure of proteins. In addition, since INDELs and VNTRs have greater potential for mutations than SNPs, they may also have an important role to play in the evolution of specific higher organism traits, such as behavior [27]. Using examples from the literature, this review highlights the functional consequences of INDELs and VNTRs in the human genome and their association with obesity and related traits. With the advent of faster and cheaper next-generation genome and exome sequencing, paired with the rise of the software availability for genome-wide detection of INDELs (like indelMINER [28]) and VNTRs (like VNTRseek [29]), it is hoped that association and functional characterization studies between INDELs/VNTRs and obesity should be continued and not neglected.

## Main text

### INDELs and VNTRs in obesity genes from the earliest GWAS and candidate gene studies

Numerous INDELs and VNTRs are located around obesity-susceptible genes or loci identified by the earliest GWAS and candidate gene studies. These mainly include genes that are involved in energy balance, appetite regulation, and adipogenesis, i.e., fat mass and obesity-associated protein gene (*FTO*), and genes in the leptin–proopiomelanocortin pathway and uncoupling protein families. It is possible that the association between INDELs/VNTRs and obesity is affected by the obesity-related SNPs because of strong linkage disequilibrium, and the risk or protective variants of INDELs/VNTRs are linked to the counterparts of the focal SNPs.

#### Fat mass and obesity-associated protein gene (*FTO*)

Genetic variants of *FTO* were the first common genetic variants to be associated with increased obesity and BMI [30], with 89 genetic variants in introns 1 and 2 that have the strongest genome-wide association signal and are in high linkage disequilibrium in Europeans [30–32]. This association was replicated in several distinct ethnic populations (reviewed in [33]), making *FTO* the single strongest genetic factor of obesity. Most studies have focused on variants of a 42-kb haplotype block around the lead SNP rs9939609 in the first intron, and recently, a causal variant rs142108 resulting in cellular phenotypes consistent with obesity, like increased triglyceride accumulation and decreased mitochondrial energy generation, has been identified [34]. *FTO* is a very large gene containing nine exons, spanning 412 kb. However, only a few studies have identified obesity-associated SNPs in other regions of the gene, in introns 2, 3 [35], and 8 [36]. By using massive parallel sequencing to sequence the entire length of *FTO*, Sällman Almén et al. identified 13 insertions and 27 deletions that range between 1 and 9 bp, of which 16 overlap known INDELs in dbSNP and 24 are potential novel INDELs [37]. Three insertions (minor allele frequency < 5%) reside within the obesity-associated haplotype of intron 1. However, none of these INDELs contributed to obesity association [37].

#### Leptin gene (*LEP*)

The discovery that the mouse obesity phenotype *ob* has been attributed to mutations in the mouse leptin gene [38], and that mutations in the human homolog (*LEP*) cause early-onset monogenic obesity in humans [6, 39], has led to significant progress in understanding the etiology of obesity. Analysis of the leptin–proopiomelanocortin (LEP–POMC) pathway has revealed the role of the pathway in hypothalamic control of feeding behavior and energy balance [40, 41]. A GWAS which identified 14 known obesity susceptibility variants and 18 new loci that were associated with BMI found that some of these loci are mapped at the LEP–POMC pathway, i.e., *LEP* and its receptor *LEPR*, *POMC* and *MC4R* [42].

While mutations in *LEP* cause monogenic obesity, there have been numerous research in determining whether genetic variations in or near *LEP* influences susceptibility to polygenic obesity. Using STR markers flanking the *LEP* locus at human chromosome 7q31.3-32.1, several groups reported evidence of linkage and/or association between these STRs and obesity-related traits, albeit with inconsistencies [43–47]. A meta-analysis of the linkage data concluded that the evidence of a gene influencing obesity in the region of the *LEP* locus was extremely strong [48]. A tetranucleotide VNTR, (CTTT)*n*, was first identified at 3912 bp 3′ of the *LEP* stop codon (476 bp 3′ of the 3′ UTR) [49]. Fifteen alleles were detected in this VNTR and were grouped into shorter class I alleles (121–145 bp) and longer class II alleles (197–225 bp); however, they were not significantly associated with obesity and T2D [49]. Nevertheless, the same group subsequently found a significant association between class I/class I genotype and hypertension, independent of obesity [50]. In another study among populations worldwide, Moffett et al. grouped this VNTR into three general classes—type 1 alleles (146–178 bp), type 2 alleles (165–193 bp), and type 3 alleles (210–254 bp), but they did not investigate the association with obesity or related traits [51]. In the Brazilian population, this VNTR was significantly associated with obesity-related traits and leptinemia, where the frequency of class I alleles was significantly higher in obese than in nonobese [52, 53]. The risk for obesity was two times higher in class I allele carriers; class I allele was associated with increased BMI and WC, and plasma leptin in women [52]. In combination with *LEP* -2548GG SNP, *LEP* VNTR/SNP class I/G combined genotypes (I/IGG, I/IGA, and I/IIGG) were significantly associated with obesity and increased BMI, WC, leptin, and triglycerides in women [53]. However, Nauruan (Pacific Islander) men homozygous for class II allele, along with homozygosity in the two other *LEPR* Gln223Arg and PRO1019pro SNPs, had elevated insulin [54]. A long-base-pair allele (class II in [49])—allele 226—of this VNTR was also associated with lower BMI in adult Samoans [55].

#### Leptin receptor gene (*LEPR*)

*LEPR*, located near the STR D1S200, has been associated with increased BMI and fat mass [56]. The *LEPR* D1S200 17 allele was associated with increased susceptibility to obesity and increased BMI, WC, and WHR in Brazilian individuals [57]. Other STRs flanking *LEPR* by approximately 9 and 3 cM, such as D1S3728 and D1S1665, were proposed to contribute to plasma leptin concentrations, adiposity, and body weight in individuals with dyslipidemia [58]. Two VNTRs located at introns 3, (CA)*n*, and 16, (CTTT)*n*, of *LEPR* were shown to be associated, respectively, with BMI and fat-free mass in the Quebec Family Study [59]. A common CTTTA-pentanucleotide 3′ UTR INDEL in *LEPR* was associated with increased body weight in patients in the Finnish Diabetes Prevention Study [60], while another novel TAT-trinucleotide INDEL variant in the 1078Y codon of *LEPR* (containing a putative phosphorylation site) was not associated with obesity in Dutch Caucasians [61].

#### Proopiomelanocortin gene (*POMC*)

A genome-wide scan found that D2S1788 GATA-tetranucleotide VNTR near *POMC* had strong linkage with serum leptin levels and fat mass in Mexican Americans [62, 63]. This VNTR was also associated with plasma leptin levels in French [64], African-American [65], and Hispanic populations [66], but not in Brazilians [57]. This VNTR has also been associated with BMI in the Framingham Heart Study families [67]. However, this VNTR together with four others near *POMC*—D2S2170, D2S144, D2S1268, and D2S1348—showed no association with obesity in Samoans [55]. Out of three VNTRs (D2S2221, D2S171, D2S2337) screened for association with obesity and related traits in French Caucasian families, only D2S2337 had linkage with serum leptin levels [68]. Lastly, a cryptic trinucleotide repeat (9-bp) INDEL detected in exon 3 of *POMC* (codon 94) [69] was associated with elevated serum leptin levels in Swedish [70] and Danish [71] men. However, this INDEL was not associated with salivary cortisol [72], obesity, and related traits [63, 71].

#### Melanocortin 4 receptor gene (*MC4R*)

Like *FTO*, *MC4R* has been extensively studied in obesity research. To date, more than 160 mutations in *MC4R*, mostly heterozygous (codominant inheritance), were identified mainly in obese individuals, encompassing nonsynonymous, nonsense, deletion, and frameshift mutations. Therefore, *MC4R* is the most commonly known monogenic cause of human obesity (reviewed in [73, 74]). In 1998, two groups reported the first functionally relevant *MC4R* mutations for obesity [75, 76]. Yeo et al. identified a hyperphagic individual who was heterozygous for a deletion of 4 bp at codon 211, resulting in a frameshift mutation which leads to a premature stop codon [75]. Another frameshift mutation (4-bp insertion after codon 244) was detected in a woman with early-onset obesity, whose family members who carried the same mutation were obese whereas the noncarriers were not obese [76]. The first mutation analysis in 306 obese individuals further confirmed the role of *MC4R* in obesity [77]. As the focus of this review is on common polygenic obesity, the examples of INDELs in *MC4R* which lead to rare monogenic obesity will not be further discussed here. The association of *MC4R* SNPs with polygenic obesity will also not be further discussed here. Nevertheless, recent GWAS have identified SNPs in the *MC4R* 3′ UTR region to have a strong association with obesity, with rs17782313 showing the second strongest association signal after *FTO* [78] and rs129070134 showing association not just in Caucasian populations but also in Asian populations [79, 80].

Eight STRs flanking the *MC4R* gene—D18S851, D18S487, D18S69, D18S858, D18S849, D18S1155, D18S64, D18S38—have demonstrated linkage with obesity in Finnish sib pairs, with D18S849 showing the strongest linkage [81]. The D18S858 11/12 allele was also associated with increased BMI and WC in Brazilian subjects [57]. This locus was also found to be related to other obesity-related complications, like systolic BP (SBP) [82] and cancer [83].

#### Uncoupling protein 2 and 3 genes (*UCP2* and *UCP3*)

Uncoupling proteins (UCPs) are mitochondrial transporters that mediate energy homeostasis and thermogenesis. Genetic variants in the *UCP2/UCP3* cluster have been considered candidate markers for obesity, diabetes, and fat metabolism in humans [84]. D11S912 and other STRs flanking *UCP2* and *UCP3* showed some evidence of linkage with obesity and BMI [4, 81]. The 43 allele of D11S912 STR was associated with increased risk for obesity in Brazilians [57], consistent with a genome-wide linkage scan study in the Framingham Heart Study families [4]. Campbell et al. reported allelic association between D11S911 and anorexia nervosa (AN) and hypothesized that this may reflect differences in metabolic rate between patients with AN and controls [85]. However, other studies found that other STRs flanking *UCP2*–*UCP3* are unlikely to have a substantial effect on the expression of obesity-related phenotypes in Mexican American [86] and Caucasian [87] populations.

A 45-bp INDEL variant in the 3′ UTR of *UCP2* exon 8 (reviewed in [88]) has been variably associated with altered BMI, changes in energy expenditure, and maintenance of body weight after overfeeding [89, 90]. Previous studies showed that carriers of the I (insertion) allele had significantly higher risk of developing obesity and BMI compared to the D (deletion) allele [88, 91–94]. In our study, we found that this INDEL was associated with obesity and overall adiposity among females [95]. However, when adjusted for age and ethnicity, this association was abolished for obesity but remained significant for overall adiposity; those carrying the ID genotype or I allele had almost twofold risk of having higher overall adiposity and had higher total body fat (TBF) compared to the D allele [95]. Similarly, Yanowski et al. also found that TBF was significantly associated and greater in ID subjects compared to II subjects [96], while WHR was not significantly different in Caucasians, African-Americans, and Asians [96]. Also, no association between this INDEL and WC and waist-to-thigh ratio was found among Pima Indians [97].

### INDELs and VNTRs in serotonergic and dopaminergic system genes

Given the important role of the brain serotonergic and dopaminergic reward system in weight gain and obesity susceptibility, investigation of the candidate genes of these neurotransmitters’ metabolism and transport is a growing area of research [98, 99].

#### 5-Hydroxytryptamine (serotonin) transporter gene (*SLC6A4*/*5HTT*)

The serotonin transporter protein (SERT or 5-HTT), which is encoded by *SLC6A4*, recycles serotonin (5-HT) after an action potential and regulates the availability of serotonin at the synaptic cleft [100]. Since its description in 1993, *SLC6A4* has been a candidate gene for a variety of neuropsychiatric conditions, given the importance of serotonergic function in mood and the widespread clinical use of selective serotonin reuptake inhibitors (SSRIs) as anxiolytics and antidepressants [100]. In addition to SNPs, two polymorphic regions influencing the transcription activity of *SLC6A4* have been studied extensively in search of association with a range of neuropsychiatric phenotypes (reviewed in [101]). The serotonin transporter-linked polymorphic region (5-HTTLPR) is located 1 kb upstream of the transcription start site and consists of a number of 20–23-bp repeat units varying from 13 to 22 units. Within the 5-HTTLPR, a 43-bp INDEL gives rise to the most common repeat elements 14R (short, S) and 16R (long, L) alleles [102]. The second 5-HTT polymorphic region is a multi-allelic 17-bp VNTR in intron 2 (named STin2) with two common alleles with 10 and 12 repeats and a rare allele with 9 repeats [103, 104].

A study with Argentinean children/adolescents demonstrated that individuals with the 5-HTTLPR S allele were found to be at greater risk of being overweight/obese as result of a genetic predisposition that leads to higher food consumption [105]. Another study with young American adults obtained similar results, whereby the prevalence of obesity combined with overweight was significantly lower among L allele carriers compared with individuals homozygous for the S allele [98]. In the same direction, Markus and Capello found that S allele carriers had significantly higher BMI than LS and LL carriers in highly neurotic individuals, indicating that cognitive stress vulnerabilities may be a mediator of the association between 5-HTTLPR and BMI [106]. In a further study by the same group, they found that SS homozygotes undergoing stress vulnerability tend to increase the appetite for sweets because sweet foods might have become more emotionally rewarding in these subjects [107]. Furthermore, Lan et al. described an age-dependent modification of 5-HTTLPR association with obesity development; they demonstrated that the SS genotype was associated with BMI and obesity in nonelderly patients with stroke but not in elderly patients [108]. Recently, it was found that Brazilian children with SS homozygotes also had higher anthropometric parameters (BMI *Z*-score, sum of skinfolds, and WC) and higher food intake at the assessments of the three stages in early childhood [109]. The S allele was also strongly associated with the presence of T2D in Greeks independent of obesity status [110], but no association with T2D and obesity was found in the Pakistani population [111].

On the other hand, Bah et al. showed different results [112]. They showed that SS tended to be more frequent in underweight subjects, and this association was significant only in Swedish men. However, there was no significant association between 5-HTTLPR and BMI [112]. Borkowska et al. detected association of the S allele with development of depressive temperament while the L allele was associated with greater obesity and prevalence of depression in Polish adults [113]. There was no significant association observed between 5-HTTLPR and BMI status in Turkish studies [114, 115].

The first study which investigated the association between STin2 VNTR and obesity found no significant effect on obesity in Turkish adults [115]. However, when combined with 5-HTTLPR, the L/10 allele haplotype showed a significant association with overweight/obesity in Portuguese adults [116]. Moreover, in inactive individuals, overweight/obesity was found nominally associated with the STin2 10 allele but significantly associated with the 5-HTTLPR L allele [116].

#### Dopamine transporter gene (*SLC6A3*/*DAT1*)

The dopamine transporter (DAT1), encoded by *SLC6A3*/*DAT1*, is a membrane-spanning protein which terminates the synaptic transmission of dopamine by reuptake into the presynaptic nerve terminals [117]. The human *DAT1* contains a VNTR in its 3′ UTR, consisting of repetitions of a 40-bp core sequence [118]. This VNTR varies between 3 and 13 repeats, of which the common 10-repeat (long; L) allele was associated with an increased expression of the transporter, leading to greater dopamine (DA) reuptake and lower synaptic levels of DA [119]. The 9-repeat (short; S) allele also has greater DA signaling in the reward circuitry than the L allele [119]. The likelihood of obesity in African-American smokers with the LL genotype was 5.16 times compared with the SS or SL genotypes [120]. This VNTR was also associated with future weight gain; S allele carriers showed greater increases in BMI than L allele carriers [121]. However, in a Turkish study, no effect of this VNTR on obesity was observed [115].

More literature reported on the association between this VNTR and eating behavior. It was found that there was an association between this VNTR and binge eating behavior [122], suggesting that dysregulation of dopamine reuptake may act as a common pathophysiological mechanism in eating disorders. It was found that smokers homozygous for the L allele and who had high food reinforcement levels consumed more energy than those with the S allele or who had low food reinforcement levels [123]. However, this finding could not be generalized to nonsmokers as the subjects were regular smokers. To circumvent this, a study conducted by the same group in a larger sample of nonsmokers did not find any significant association between this VNTR and energy intake or between VNTR and food reinforcement [124]. Results also supported the role of DAT1 in regulating appetitive response to methylphenidate, a psychomotor stimulant. Subjects with binge eating disorders with at least one S allele showed a significant suppression of appetite in response to methylphenidate, compared with controls with this allele or to subjects with the LL genotype [125].

#### Dopamine receptor D2 gene (*DRD2*)

Obese subjects, relative to lean ones, have reduced D2 receptors (DRD2) in the striatum [126], due to decreased metabolism in prefrontal cortical regions [127]. An inverse relationship between BMI and DRD2 has also been described; specifically, those individuals with the lowest DRD2 brain density had the largest BMI [126]. In a single study examining the association between *DRD2-141C* INDEL and future weight gain, there was no association found between this INDEL and change in BMI over a 2-year follow-up [121]. Other studies tend to investigate the association of another genetic variant that modulates the density of DRD2—*DRD2*/*ANKK1*-TaqIA SNP—with both obesity [126] and addictive disorders [128, 129] instead, which is out of the scope of this review.

#### Dopamine receptor D4 gene (*DRD4*)

Another gene encoding dopamine receptors is *DRD4*, which is highly expressed in the prefrontal cortex and other brain regions that are involved in the reward circuits that mediate the reinforcing properties of food, such as the amygdala, hippocampus, and hypothalamus [130, 131]. This gene contains a highly polymorphic 48-bp VNTR within exon 3, which is located in the third cytoplasmic loop of the receptor [132]. Nine alleles of this VNTR have been identified worldwide, with the number of repeats ranging from 2 to 10; 4-repeat (4R; S) and 7-repeat (7R; L) alleles are the most common [133]. This VNTR has been hypothesized to affect the transmitted signal in the postsynaptic neuron. Individuals with at least one ≥7R allele showed reduced binding affinities and receptor densities for dopamine neurotransmission [134]. This VNTR has also been associated with increased food intake in patients treated with DRD4-related antipsychotics [135], and with addictive behaviors [136]. In some studies, *DRD4* VNTR and higher BMI correlation was shown [137, 138]. The S allele was also associated with increases in BMI over a 2-year follow-up period [121].

*DRD4* VNTR also influences BMI and body composition in the context of other environmental factors. For example, 7R allele/season-of-birth interactions increase the risk for obesity in women with either seasonal affective disorder [139] or bulimia nervosa [140]. A study of Kenyan Ariaal men found that BMI was higher in those with one or two 7R alleles in the nomadic population, but lower among the settled, due primarily to differences in fat-free body mass [141]. Children who carried the 7R allele also appeared to be more influenced by maternal sensitivity (response to a child’s emotional signals) as it relates to overweight/obesity risk [142]. However, there was no association between this VNTR alleles with AN, underweight, or extreme early-onset obesity [143]. Similarly, no association was detected between alleles/genotypes and BMI, BMI-SDS, or skinfold thickness at baseline nor success in the weight loss intervention in obese German children [144]. Similarly, there was no association found with obesity in Turkish adults [115]. There was also no association found between level of physical activity and sedentary lifestyle in Polish adults [145].

#### Monoamine oxidase A gene (*MAOA*)

Monoamine oxidase (MAO) is an outer membrane mitochondrial enzyme that catalyzes the turnover of several catecholamine neurotransmitters, namely serotonin, noradrenaline, and dopamine [146]. MAOA, an isoenzyme of MAO, is encoded by *MAOA* located on the short arm of the X chromosome between bands Xp11.23 and Xp11.4 [147]. It contains a 30-bp VNTR located at the promoter region, manifesting as six functional allele variants containing either 2-, 3-, 3.5-, 4-, 5-, or 6-repeat copies [146], with the 3- (3R) and 4-repeat (4R) alleles being the most common [148]. Certain alleles may confer lower transcriptional efficiency than others; the 3R allele conveys lower efficiency, the 3.5-repeat and 4R alleles result in higher efficiency [146, 149], while to date, there is less consensus regarding the transcriptional efficiency of the other less commonly occurring alleles (i.e., 2-, 5-, and 6-repeat). The primary role of MAOA in regulating catecholamine turnover and hence ultimately influencing their levels indicates that its VNTR is a highly plausible candidate for affecting individual differences in physiological and psychological traits, such as obesity, personality traits, and alcoholism risk [150]. A previous study showed a significant association of *MAOA* VNTR alleles (either alone or with combination of other gene variants) with weight and BMI in Caucasian populations [98, 151–153], while our unpublished study showed association only with weight; both studies agree that subjects having the lower activity 3R allele tend to have a lower weight than the higher activity 4R allele. Meanwhile, another study showed a significant association of this VNTR with obesity in a mixed-ethnic American population [154], while Dias et al. [116] and our unpublished studies showed otherwise. In a previous study, Brazilian boys who carried the 4R allele were associated with higher intake of lipid- and sugar-dense foods [155]. The increased intake of lipid- and sugar-dense foods might have led to increased lipid accumulation in adipose tissues, leading to higher TBF and SF in 4R allele carriers, as shown in our unpublished study. However, a previous study showed that Portuguese men with the 3R3R genotype had significantly higher TBF [116].

#### Tyrosine hydroxylase gene (*TH*)

Tyrosine hydroxylase (TH) is a rate-limiting enzyme in adrenaline, noradrenaline, and dopamine synthesis, and its coding gene, *TH*, is located at chromosome 11p15 [156]. An extensively studied TH polymorphism known as (TCAT)_*n*_ is a tetranucleotide VNTR located in the first intron of *TH*, giving five alleles—T6, T7, T8, T9, and T10 [157]. The alternate splicing process of the (TCAT)_*n*_ polymorphism involving the 3′ end of exon 1 and differential exclusion at exon 2 will produce an uneven regulatory effect due to the different combination of (TCAT)_*n*_ repeats [158]. It has also been reported that the cerebrospinal fluid and serum concentrations of the metabolites in the dopaminergic pathway of humans have been altered by this VNTR [159, 160]. Since TH is the rate-limiting enzyme for the biosynthesis of catecholamines, this VNTR has attracted considerable attention and is a candidate marker for various neuropsychiatric phenotypes like schizophrenia, mood disorders, alcohol dependence [161], and personality traits [162]. However, the association of this polymorphism with obesity is still elusive as there is scarce data on it. *TH* mRNA expression was significantly decreased in the substantia nigra, ventromedial hypothalamic nucleus, and ventral tegmental area of the high-fat-diet-induced obese and obese-resistant mice compared to controls [163]. Individuals with central obesity had lower *TH* expression in their peripheral blood mononuclear cells (PBMCs) compared with controls, and *TH* expression was also significantly negatively correlated with WC [164]. In our unpublished study, we found that Malaysian subjects with *TH* VNTR was not associated with obesity, but nevertheless, subjects with the T9 allele had significantly highest SBP and visceral fat level (VFL) and lowest pulse rate.

### VNTR in insulin gene (*INS*)

Free fatty acid accumulation in the liver, adipose tissue, and skeletal muscle of obese patients interferes with normal insulin signaling, which will lead to insulin resistance [165]. As a consequence, increased insulin production (hyperinsulinemia) in the pancreas is needed to maintain normal values of glycemia, which, in turn, converts the liver into a fat-producing factory with all of its negative downstream effects [165].

A gene that may be involved in the development of metabolic syndrome is the gene codifying for insulin itself (*INS*). *INS* is located between *TH* and the insulin-like growth factor II gene (*IGF2*) on 11p15.5 [166]. Within this gene, a VNTR, positioned at 363 bp from the *INS* transcription starting site (promoter region), has been largely studied in cohorts of children and adolescents [167, 168]. Three alleles of *INS* VNTR have been observed in Caucasians: the common short class I (26–63 repeats) and long class III (141–209 repeats) and the rare class II (about 80 repeats) [168, 169]. Differences in steady-state levels of *INS* mRNA have been detected in the pancreas of a cadaver adult and fetus carrying class I and class III alleles, albeit with lower levels in the latter allele [170, 171].

The *INS VNTR* has been intensively studied for association with glucose metabolism in a number of metabolic disorders. Initially, class I allele was reported to be associated with higher risk of type 1 diabetes in UK families [168], and this finding has been consistently and reliably replicated [172]. On the other hand, class III allele was suggested to associate with higher birth weight [173], higher adult fasting glucose [174] and infant insulin [175] levels, and increased risk of T2D [176]. However, several studies failed to replicate association of class III allele with fetal growth and birth weight [177, 178] or reported associations with lower birth weight [174, 179]. Findings on the *INS* VNTR association with T2D were also mainly not successfully replicated [180, 181], although a modest association was reported in a family-based study [182].

The putative contribution of the *INS* VNTR in the genetic risk for obesity was first investigated in children. Strong evidence for a family-based association with 1.8-fold increased risk of early-onset obesity for class I allele was found, when this allele is paternally inherited [183]. An earlier study by the same group has suggested higher insulin levels in obese children carrying the class I allele [171]. However, several studies of the association of *INS* VNTR with body composition and insulin levels in cohorts of children were inconclusive [184–187]; for example, large Finnish birth cohort [180] and UK population of middle-aged adults [188] studies failed to find any significant association. The relevance of studies employing unrelated individuals has been criticized [189], due to heredity complexity and transmission distortion of the *INS* VNTR [190]. However, in a family-based design in more than 1000 French or German Caucasian families, this VNTR was not associated with childhood obesity and variance of insulin resistance, insulin secretion, and birth weight [191].

### VNTRs in inflammatory cytokine genes

#### Interleukin-1 receptor antagonist gene (*IL1RA*)

Interleukin-1 receptor antagonist (IL-1ra), also known as IL-1RN, is an endogenous competitive inhibitor of proinflammatory IL-1α and IL-1β [192]. IL-1ra is a proadipogenic factor as it is highly secreted by the white adipose tissue [193], and IL1RA knockout mice have impaired adipogenesis and reduced adipose storage [194]. The IL-1ra level is increased in the serum of obese patients, correlating with BMI and insulin resistance [195]. A 86-bp VNTR is found within intron 2 of *IL1RA*, and to date, six distinct alleles corresponding to 1, 2, 3, 4, 5, and 6 copies of the VNTR have been identified [196]. The 4-repeat (allele I) and 2-repeat (allele II) are most frequently found in the general population, while the other four (alleles III, IV, V, and VI) are rarely observed [197]. This VNTR, particularly homozygosity for allele II, has been variably associated with various conditions such as obesity, inflammatory bowel disease, and coronary artery disease (reviewed in [198]). IL1RA allele II has a clear influence on IL-1ra circulating levels since in normal human subjects, its carrier had higher levels than the noncarrier individuals [199]. With regard to obesity, two previous Asian studies with relatively small sample sizes found no significant association between *IL1RA* VNTR and BMI in Koreans [200] and North Indians [201]. Similarly, our study found no association with BMI value or overall obesity status, but *IL1RA* II allele VNTR was associated with higher TBF value and higher risk for overall adiposity [202].

#### Interleukin-4 gene (*IL4*)

IL-4, secreted by activated Th2 lymphocytes, basophils, and mast cells, executes many biological roles such as induction of Th2 differentiation, B cell proliferation, and immunoglobulin class switching [203]. In animal studies, mice treated with IL-4 had improved insulin sensitivity and glucose tolerance while lipid accumulation in adipose tissues was inhibited [204, 205], while rats receiving visceral fat removal surgery had decreased serum IL-4 [206]. The role of IL-4 in modulating adipogenesis has been established by previous studies [207, 208]. Similar to *IL1RA*, *IL4* has a 70-bp VNTR polymorphism within intron 3, and two common alleles are B1 and B2 that have two and three tandem repeats, respectively [209]. This VNTR could be a functional polymorphism as it could affect mRNA splicing, leading to different splice variants [210]. Indeed, the B2 allele has been associated with a reduced amount of peripheral Th cells which produce IL-4 [211]. There have been several reports on the association between the VNTR B1 allele and inflammatory diseases, such as multiple sclerosis [212], rheumatoid arthritis [213], and systemic lupus erythematosus [214]. With regard to obesity, there are limited studies on this VNTR, where two studies showed no association [215, 216]. Our study showed that this VNTR was associated with overall adiposity status (TBF class), but not with TBF value after adjustment for ethnicity [202]. A previous study in north Indians also showed that the B2B2 genotype was associated with higher risk for T2D [217].

### Other INDELs and VNTRs

#### Endothelial nitric oxide synthase gene (*eNOS*/*NOS3*)

Nitric oxide (NO) is synthesized from l-arginine by nitric oxide synthase (NOS). There are at least three isoenzymes of NOS: constitutive neuronal NOS (nNOS or NOS-1), inducible NOS (iNOS or NOS-2), and constitutive endothelial NOS (eNOS or NOS-3), located on different chromosomes and expressed in different cell lines [218]. eNOS has been described as a major regulator of adipose tissue metabolism and energy balance by affecting lipolysis [219]. The adipose tissue and skeletal muscle of obese humans and rodents have decreased eNOS [220–222].

*eNOS* has been reported to possess approximately 303 variations including a VNTR at intron 4, intron 13 with a CA repeat, and enormous SNPs. There are three variations which are putatively functional and studied frequently, namely Glu298Asp, 27-bp VNTR intron 4a/b, and T-786C [223]. At the 27-bp VNTR, a larger (b) allele comes with five tandem repeats while a smaller (a) allele comes with four tandem repeats [223]. Plasma NO levels were found to be significantly lower in individuals homozygous for the (b) allele than in those homozygous for the (a) allele, suggesting a role of this VNTR in the regulation of *eNOS* expression [224, 225]. Indeed, this VNTR might have a *cis*-acting role in *eNOS* promoter function [226]. Others revealed that this VNTR produces small microRNAs that induce significant *eNOS*-specific transcriptional suppression by altering histone acetylation and DNA methylation [227]. Specifically, the (b) allele could result in increased microRNA expression and reduced *eNOS* mRNA levels [228]. Overall, these studies highlight that the (b) allele correlates with reduced *eNOS* expression and reduced NO levels, which may lead to low fat oxidation over time and a mild progression of increase in body fat [229].

A pioneer study investigating the effect of this VNTR on obesity in a sample of the Tunisian population found that carriers of the (b) allele presented 1.7 times higher risk of obesity [230]. Correlations with anthropometric parameters also revealed that carriers of the bb genotype had significantly higher BMI compared to those homozygous for the (a) allele [231]. Although this VNTR has not been reported to be associated with T2D, other *eNOS* SNPs are associated with T2D [231] and insulin resistance [232–234]. *eNOS* SNPs also appear to increase susceptibility for hypertriglyceridemia and low HDL [235] and worsen endothelial function in individuals prone to T2D [236]. However, neither the *eNOS* VNTR allele, genotype, nor their combination with angiotensin-converting enzyme (*ACE*) INDEL, apolipoprotein E (*APOE* ε2, ε3, ε4), and *LEP* G2548A presented as a risk for hypertension, elevated triglycerides, and total, LDL, or HDL cholesterol in the Roma minority population of Croatia [237].

#### Angiotensin-converting enzyme gene (*ACE*)

ACE, a key enzyme in the renin–angiotensin system (RAS), converts angiotensin I into vasoconstrictor molecule angiotensin II [238]. Body fat and body weight could be raised and lowered accordingly by stimulating and inhibiting the production of Ang II [239], suggesting a possible link between ACE and obesity (reviewed in [240]). In 1990, Rigat et al. first described an INDEL in *ACE*, defined as either the presence (insertion, I) or absence (deletion, D) of a 287-bp insert in intron 16 of the gene on chromosome 17q23 (dbSNP rs1799752) [241]. This INDEL has been proposed as a genetic marker for a variety of disease conditions (reviewed in [242]), ranging from mainly cardiovascular diseases like essential hypertension [243], myocardial infarction [244], and hypertrophic cardiomyopathy [245] to obesity-related complications such as metabolic syndrome [246], T2D [247], and reduced HDL-C levels [248].

Research on the association of this INDEL with obesity specifically has been growing in the past two decades, mainly showing the role of the D allele or DD genotype in increased risk for overweight/obesity. The DD genotype was associated with larger increases in body weight and BP in older Italian men, as well as with higher incidence of overweight [249]. Spanish subjects with coronary heart disease (CHD) and DD/ID genotypes had significantly higher prevalence of obesity and abdominal fat deposit and higher values of weight and WC [250]. The DD genotype and D allele occurred at a higher frequency in obese Saudi [251] and Turkish [252] individuals. The D allele was associated with increased BMI, WC, and body fat mass in Brazilian boys, independent of the association with BP [253]. The DD genotype also heightened the effects of traditional risk factors for obesity, for example by increasing the magnitude of the association between childhood gain in upper body adiposity, insulin resistance, and hypertriglyceridemia [254] and by increasing carbohydrate intake in morbidly obese Czech population [255]. In contrast, our study found that Malaysian subjects with the II genotype and I allele were, respectively, 2.15 and 1.55 times more likely to be centrally obese, but when adjusted for age and ethnicity, this association was abolished [256]. However, other studies reported that this INDEL was not associated with obesity-related traits in overweight sedentary American women [257], Korean women [258, 259], and a large sample of Chinese patients with T2D [260].

#### Dedicator of cytokinesis 5 gene (*DOCK5*)

In-depth investigation of a complex region on chromosome 8p21.2 encompassing *DOCK5* which includes two VNTRs of complex sequence composition (one in 5′ UTR and another in intron 1 of *DOCK5*) which flank a common 3975-bp INDEL (in intron 1 of *DOCK5*) found a significant association of these VNTRs and INDEL with childhood and adult severe obesity [261]. Support for a functional effect of the *DOCK5* VNTRs and deletion was also evidenced by the association between *DOCK5* transcript levels and variants in adipose tissue from a Swedish family sample [261]. The mechanism of how *DOCK5* could contribute to obesity is currently unknown, but it has been postulated that DOCK5, a member of the DOCK family of guanine nucleotide exchange factors, could bind to protein phosphatase 2 to inactivate v-akt murine thymoma viral oncogene homolog (Akt) proteins and mitogen-activated protein kinases 1 and 3 (MAPK1 and 3), modulating the anorectic effects of leptin [261].

#### Period circadian clock 3 gene (*PER3*)

A 54-bp VNTR in exon 18 of *PER3* defined as four repeats (4 allele) or five repeats (5 allele) in humans [262] has been associated with sleep behavior [263]. Particularly, the *PER3* 5/5 genotype was associated with increased morning preference, earlier wake and bed time, and reduced daytime sleepiness [264]. In a single study, this VNTR has been investigated for association with obesity-related anthropometric traits, sleep, and nutritional behavior [265]. No association with obesity was found. Nevertheless, individuals with the 55 genotype had a higher percentage of daily energy derived from fat, had a lower percentage of daily energy derived from carbohydrates, and were more prone to an age-dependent increase in cholesterol intake [265].

## Points of concern

A review paper on the role of INDELs and VNTRs in obesity is virtually nonexistent. Hence, this paper reviews the types of INDELs and VNTRs that have been studied for association with obesity and its related traits and complications, as summarized in Table 1. Some studies showed significant associations between INDELs/VNTRs and obesity-related traits whereas other studies were not the case.

**Table 1:** Summary of INDELs and VNTRs that have been studied for association with common polygenic obesity and its related traits and complications

Gene/nearest gene	INDEL/VNTR	Type(s) [pivotal reference]	Location in human chromosome
*FTO*	INDELs	13 insertions and 27 deletions (1–9 bp)—16 known in dbSNP and 24 potentially novel [37]	hg18, chr16: 52307514–52699069
*LEP*	VNTR	(CTTT)*n* Type 1 allele: 121–145 [49] or 146–178 bp [51] Type 2 allele: 197–225 [49] or 165–193 bp [51] Type 3 allele: 210–254 bp [51]	3912 bp 3′ of the *LEP* stop codon (476 bp 3′ of the 3′ UTR)
*LEPR*	STR/VNTR	D1S200: (CA)*n*; 13–27 repeats; 17 most common [56, 57]	UniSTS: 56325; Chr1 c.77.73 cM
		D1S3728 [58]	UniSTS: 56029; Chr1 c.89.49 cM
		D1S1665 [58]	UniSTS: 60783; Chr1 c.99.62 cM
		(CA)*n* [59]	Intron 3 of *LEPR*
		(CTTT)*n* [59]	Intron 16 of *LEPR*
	INDEL	CTTTA [60]	3′ UTR of *LEPR*
		TAT [61]	1078Y codon of *LEPR*
*POMC*	STR/VNTR	D2S1788: (GATA)*n*; 4–20 repeats; 15 most common [62, 63]	UniSTS: 6210; Chr2 55.51 cM
		D2S2170 [55]	UniSTS: 60770; Chr2 47.98 cM
		D2S144 [55]	UniSTS: 68025; Chr2 45.3 cM
		D2S1268 [55]	UniSTS: 149288; Chr2
		D2S1348 [55]	UniSTS: 54913; Chr2
		D2S2221 [68]	UniSTS: 32562; Chr2 46.54 cM
		D2S171 [68]	UniSTS: 73301; Chr2 149.4 cr3000
		D2S2337 [68]	UniSTS: 14003; Chr2 47.76 cM
	INDEL	Trinucleotide repeat (9 bp) [69]	Exon 3 of *POMC* (codon 94)
*MC4R*	STR/VNTR	D18S851 [81]	UniSTS: 39301; Chr18 382.9 cr3000
		D18S487 [81]	UniSTS: 84391; Chr18 20924 cr50000
		D18S69 [81]	UniSTS: 47737; Chr18 2049 cr10000
		D18S858 [81]; 8–20 repeats; 11/12 most common [57]	UniSTS: 14041; Chr18 80.41 cM
		D18S849 [81]	UniSTS: 15592; Chr18 430.2 cr3000
		D18S1155 [81]	UniSTS: 32047; Chr18 81.27 cM
		D18S64 [81]	UniSTS: 17561; Chr18 84.8 cM
		D18S38 [81]	UniSTS: 14742; Chr18 84.98 cM
*UCP2*	STR/VNTR	D11S912 [4, 57, 81]	UniSTS: 72663; Chr11 137.93 cM
	INDEL	45 bp [88]	3′ UTR of *UCP2* exon 8
*SLC6A4/5HTT*	VNTR	5-HTTLPR 43 bp; 14R (short, S) and 16R (long, L) alleles [102]	1 kb upstream of the *SLC6A4/5HTT* transcription start site
		STin2 17 bp; common 10- and 12-repeat alleles and a rare 9-repeat allele [103, 104]	Intron 2 of *SLC6A4/5HTT*
*SLC6A3/DAT1*	VNTR	40 bp; 9-repeat (S) and 10-repeat (L) alleles [118]	3′ UTR of *SLC6A3/DAT1*
*DRD2*	INDEL	1 bp; dbSNP rs1799732; -141C [121]	5′ UTR of *DRD2*
*DRD4*	VNTR	48 bp; 2–10 repeats; 4-repeat (4R or S) and 7-repeat (7R or L) alleles most common [132]	Exon 3 of *DRD4*
*MAOA*	VNTR	30 bp; 2-, 3-, 3.5-, 4-, 5-, or 6-repeat copies [151]; 3- and 4-repeat alleles most common [146]	Promoter region of *MAOA*
*TH*	VNTR	(TCAT)_*n*_; T6, T7, T8, T9, and T10 alleles [157]	Intron 1 of *TH*
*INS*	VNTR	14–15 bp; short class I allele (~570 bp, 26–63 repeats), class II alleles (~1320 bp, about 80 repeats), and the long class III (~2470, 141–209 repeats) [167]	63 bp from the *INS* transcription starting site (promoter region)
*IL1RA*	VNTR	86 bp; 1, 2, 3, 4, 5, and 6 repeats; 4-repeat (allele I) and 2-repeat (allele II) most common [196]	Intron 2 of *IL1RA*
*IL4*	VNTR	70 bp; 2-repeat (B1) and 3-repeat (B2) alleles most common [209]	Intron 3 of *IL4*
*eNOS/NOS3*	VNTR	27 bp; 4-repeat (a) and 5-repeat (b) alleles most common [223]	Intron 4 of *eNOS/NOS3*
*ACE*	INDEL	287 bp; dbSNP rs1799752 [241]	Intron 16 of *ACE*
*DOCK5*	VNTR	VNTR A; 590–640-bp allele [261]	chr8: 25085372–25085875; ~12 kb upstream of *DOCK5*
		VNTR B; 944–1022-, 1112–1127-, 1073–1084-, and 1099–1103-bp alleles [261]	chr8: 25129579–25130501; intron 1 of *DOCK5*
	INDEL	3975 bp [261]	chr8: 25122602–25126576; intron 1 of *DOCK5*
*PER3*	VNTR	54 bp; 4-repeat and 5-repeat alleles [262]	Exon 18 of *PER3*

Before the establishment of SNP-based GWAS genotyping platforms like SNP microarray, INDELs and VNTRs were commonly used as a genetic maker for linkage and candidate gene association analyses of polygenic traits including obesity, as evidenced by the numerous obesity sequence tagged sites (STSs) as stated in the earlier part of the review. This is because genotyping of INDELs and VNTRs is easy with low-resolution gel electrophoresis methods. Virtually, obesity loci identified in the past studies using INDELs or VNTRs were not replicated in the SNP-based GWAS with enough sample sizes. It suggests that the many of old-fashioned obesity loci were unfortunately false positive. Besides insufficient sample size, these false positive loci could also be attributed to the other reasons as outlined by Li and Meyre [10], which include nonheritable phenotype, improper correction for multiple testing, population stratification, technical biases, insufficient quality control, or inappropriate statistical analyses.

Even if an original study describes a true positive association for a particular INDEL/VNTR, replication could be challenging, as evidenced by the discrepancies of findings in the association studies described in this review. Again, the reasons as outlined by Li and Meyre [10] include underpowered replication samples, interaction between genes themselves and with the environment, genetic heterogeneity (due to ethnicity), phenotypic heterogeneity (different definitions, measurements, and categorizations for obesity), and subjective interpretation of data.

Some of these pitfalls in obesity loci discovery and replication genetic association studies could be overcome by following the recommendations by Li and Meyre [10] and the STrengthening the Reporting of OBservational studies in Epidemiology - Molecular Epidemiology (STROBE-ME) statement [266].

## Conclusions

INDELs and VNTRs have significant functional consequences by regulating gene transcription, translation efficiency, and stability of mRNA or by modifying the activity of proteins by altering their structure. The final publication of the phase 3 1000 Genomes Project in October 2015, which has 3.4 million bi-allelic INDELs and 60,000 structural variants [267], has provided a marker set for the imputation of genotypes in recent GWAS. Common INDELs and VNTRs that are in the promoters and exons or have been studied before in previous candidate gene association studies could be highly prioritized in the candidate gene approach in finding obesity loci. INDELs and VNTRs would greatly expand the number of high-scoring variants besides SNPs that are identified in obesity candidate gene studies and GWAS. As an association does not always imply causality, biological insights are essential in increasing the credibility of the observed genetic association. Therefore, in silico, in vitro, and ex vivo functional characterization assays of the INDELs and VNTRs could then be performed, especially for novel genes, to elucidate the mechanistic effects of the risk alleles in plausible biological pathways involved in obesity. This could be followed by comprehensive in vivo animal studies to ultimately identify the risk allele as a causal variant for obesity and/or its related traits and complications. Inclusion of INDELs and VNTRs in genetic association studies would help in defining the genetic architecture of complex traits and diseases like obesity and also to provide new insights into its normal physiology and disease pathophysiology. Identification of the causal relationships between INDELs and VNTRs and obesity risk would facilitate the prediction of obesity onset, early diagnosis of obesity, and the development of novel and potentially patient-specific therapeutic targets.

## Abbreviations

5-HT: Serotonin
AN: Anorexia nervosa
BMI: Body mass index
bp: Base pair
BP: Blood pressure
CNV: Copy number variation
DA: Dopamine
*DOCK5*: Dedicator of cytokinesis 5 gene
*DRD2*: Dopamine receptor D2 gene
*DRD4*: Dopamine receptor D4 gene
*eNOS/NOS3*: Endothelial nitric oxide synthase gene
*FTO*: Fat mass and obesity-associated protein gene
GIANT: Genetic Investigation of Anthropometric Traits
GWAS: Genome-wide association study
HDL-C: High-density lipoprotein cholesterol
*IL1RA*: Interleukin-1 receptor antagonist gene
*IL4*: Interleukin-4 gene
INDEL: Insertion/deletion
*INS*: Insulin gene
*LEP*: Leptin gene
*LEPR*: Leptin receptor gene
LDL: Low-density lipoprotein
LOF: Loss-of-function
*MAOA*: Monoamine oxidase A gene
*MC4R*: Melanocortin 4 receptor gene
NO: Nitric oxide
NOS: Nitric oxide synthase
*PER3*: Period circadian clock 3 gene
*POMC*: Proopiomelanocortin gene
RAS: Renin–angiotensin system
SBP: Systolic blood pressure
SF: Subcutaneous fat
*SLC6A3/DAT1*: Dopamine transporter gene
*SLC6A4/5HTT*: 5-Hydroxytryptamine (serotonin) transporter gene
SNP: Single nucleotide polymorphisms
STR: Short tandem repeat
STS: Sequence tagged site
T2D: Type 2 diabetes
TBF: Total body fat
*TH*: Tyrosine hydroxylase gene
*UCP2*: Uncoupling protein 2 gene
*UCP3*: Uncoupling protein 3 gene
VFL: Visceral fat level
VNTR: Variable number tandem repeat
WC: Waist circumference
WHR: Waist–hip ratio
